# A cluster of three extrapulmonary *Mycobacterium abscessus* infections linked to well-maintained water-based heater-cooler devices

**DOI:** 10.1017/ice.2023.273

**Published:** 2024-05

**Authors:** Jessica L. Seidelman, Arthur W. Baker, Sarah S. Lewis, Bobby G. Warren, Aaron Barrett, Amanda Graves, Carly King, Bonnie Taylor, Jill Engel, Desiree Bonnadonna, Carmelo Milano, Richard J. Wallace, Matthew Stiegel, Deverick J. Anderson, Becky A. Smith

**Affiliations:** 1 Duke Center for Antimicrobial Stewardship and Infection Prevention, Durham, North Carolina; 2 Division of Infectious Diseases, Duke University Medical Center, Durham, North Carolina; 3 Disinfection, Resistance, Transmission and Epidemiology Laboratory, Department of Medicine, Duke University Medical Center, Durham, North Carolina; 4 Division of Cardiovascular and Thoracic Surgery, Duke University School of Medicine, Durham, North Carolina; 5 Mycobacteria/Nocardia Laboratory, University of Texas Health Science Center, Tyler, Texas; 6 Occupational and Environmental Safety Office, Laboratory Safety, Duke University and Health System, Durham, North Carolina

## Abstract

**Background:**

Various water-based heater-cooler devices (HCDs) have been implicated in nontuberculous mycobacteria outbreaks. Ongoing rigorous surveillance for healthcare-associated *M. abscessus* (HA-Mab) put in place following a prior institutional outbreak of *M. abscessus* alerted investigators to a cluster of 3 extrapulmonary *M. abscessus* infections among patients who had undergone cardiothoracic surgery.

**Methods:**

Investigators convened a multidisciplinary team and launched a comprehensive investigation to identify potential sources of *M. abscessus* in the healthcare setting. Adherence to tap water avoidance protocols during patient care and HCD cleaning, disinfection, and maintenance practices were reviewed. Relevant environmental samples were obtained. Patient and environmental *M. abscessus* isolates were compared using multilocus-sequence typing and pulsed-field gel electrophoresis. Smoke testing was performed to evaluate the potential for aerosol generation and dispersion during HCD use. The entire HCD fleet was replaced to mitigate continued transmission.

**Results:**

Clinical presentations of case patients and epidemiologic data supported intraoperative acquisition. *M. abscessus* was isolated from HCDs used on patients and molecular comparison with patient isolates demonstrated clonality. Smoke testing simulated aerosolization of *M. abscessus* from HCDs during device operation. Because the HCD fleet was replaced, no additional extrapulmonary HA-Mab infections due to the unique clone identified in this cluster have been detected.

**Conclusions:**

Despite adhering to HCD cleaning and disinfection strategies beyond manufacturer instructions for use, HCDs became colonized with and ultimately transmitted *M. abscessus* to 3 patients. Design modifications to better contain aerosols or filter exhaust during device operation are needed to prevent NTM transmission events from water-based HCDs.

Water-based heater-cooler devices (HCDs) used in cardiothoracic (CT) surgeries have been implicated as a point source for numerous extrapulmonary nontuberculous mycobacteria (NTM) outbreaks around the world.^1–4^ Accordingly, the US Food & Drug Association (FDA) continues to work with manufacturers to validate cleaning and disinfection protocols and now requires aerosol testing.^5^

Baker et al^6^ previously described a 2-phase clonal outbreak of *Mycobacterium abscessus* linked to in-hospital tap water exposure at our hospital. Since the outbreak, strict protocols for tap water avoidance during clinical care of epidemiologically relevant patient populations; meticulous cleaning, disinfection, and maintenance practices for the HCD fleet; and ongoing surveillance for hospital-acquired. *M. abscessus* (HA-Mab) infections have continued with high reliability.

In June 2018, a multidisciplinary team proactively convened to perform a risk assessment of all HCD models available in the United States. The team ultimately recommended replacing the hospital’s fleet of LivaNova 3T HCDs (LivaNova, Houston, TX) with CardioQuip MCH(i)-1000 HCDs (CardioQuip, College Station, TX) given the prior institutional experience and limited understanding of the efficacy of the LivaNova 3T HCD design modifications and other mitigation strategies at that time. Following the product conversion, the team recommended using strongest disinfectant (bleach) among those listed in the manufacturer’s instructions for use (IFU) as well as an increased frequency for deep cleaning (monthly vs quarterly). Despite robust adherence to the aforementioned protocols, a cluster of HA-Mab infections involving 3 CT surgery patients who underwent surgery at our hospital between October and December of 2020 was detected, prompting investigation and mitigation.

## Methods

### Study setting

Duke University Hospital (DUH) is a 1,048-bed quaternary-care hospital in Durham, North Carolina. DUH is a high-volume surgical center where ∼1,700 CT surgeries are performed annually, including >250 heart and lung transplant surgeries per year.

The Duke University Institutional Review Board approved this investigation and research.

### Epidemiologic investigation

Investigators defined a case of HA-Mab infection as a patient with a first-time positive culture for *M. abscessus* after hospital day 2, a patient who had been hospitalized at DUH within 30 days prior to culture collection, or a patient with a surgical site infection due to *M. abscessus* on whom the index surgery had been performed at DUH. Monthly surveillance for HA-Mab infections was performed, and detailed chart review was completed for all potential HA-Mab cases.

Although routine surveillance included all HA-Mab infections, for the purposes of this investigation, investigators focused on patients who underwent a CT surgery at DUH with extrapulmonary infection meeting the case definition. Incidence rates of HA-Mab infections in CT surgery patients from the previous outbreak period July 2013 through December 2015 (period 1) were compared to 3 subsequent periods: period 2 (January 2016–September 2020), period 3 (October 2020–December 2020) and period 4 (January 2021–December 2022). Periods were delineated based on HA-Mab incidence rate data. Incidence rates of HA-Mab surgical site infections, limited to positive cultures from sterile body sites (blood and tissue) for the same time periods were also compared. The index CT surgery date was used as the date of infection, given the variable time from inoculation to clinical presentation and diagnosis, and the focus on potential intraoperative sources of inoculation.

### Field investigation

**Figure 1.**
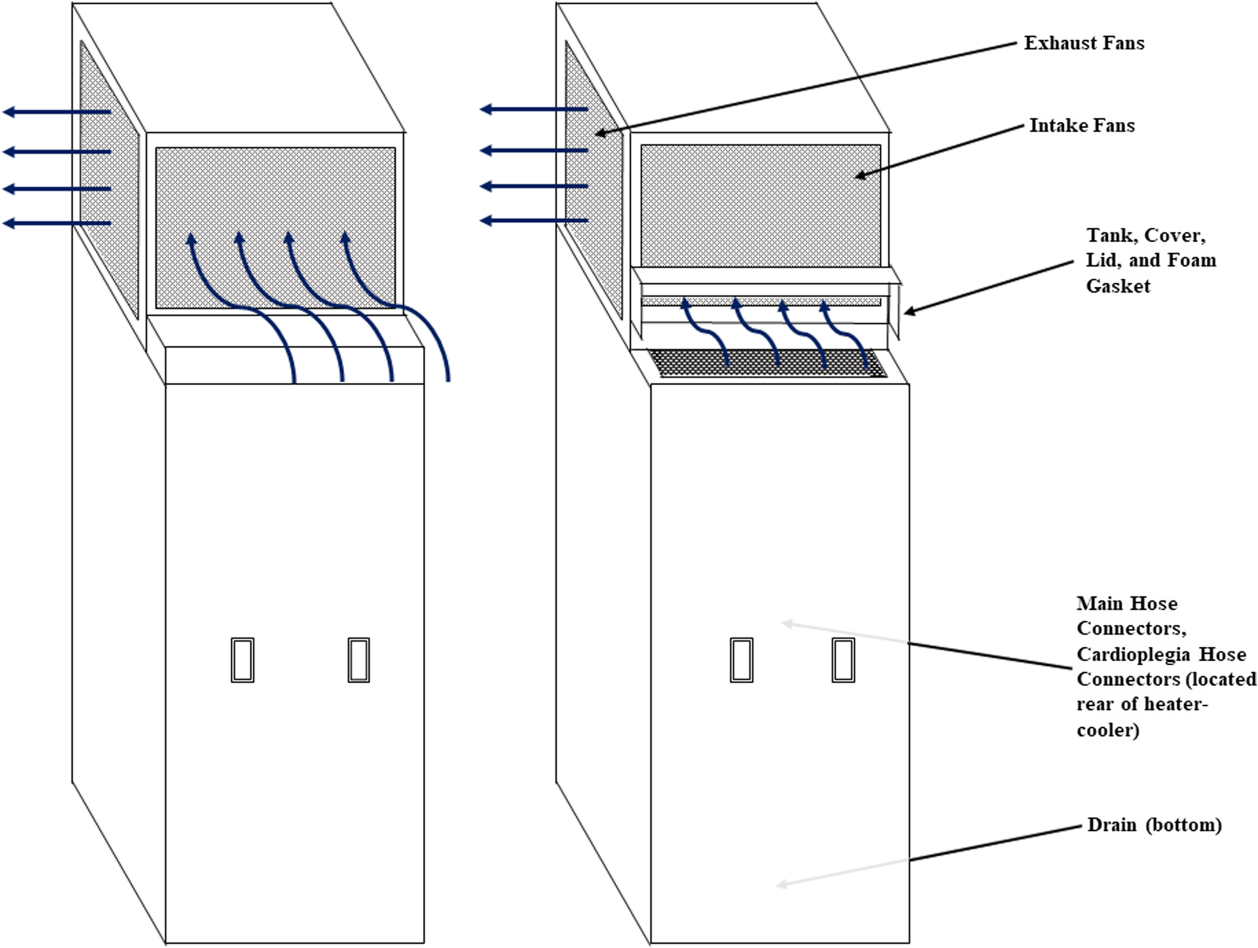
Diagram of the Cardioquip heater-cooler device durign smoke testing and specific locations of the environmental cultures. In scenario A, the water-check door on the “front” is closed and the application of micro puff smoke at various locations around the top of the unit results in an airflow pattern where air enters the “front” and exits through the sides. In scenario B, the water-check door is open and the application of micro puff smoke generation in the water tank demonstrates an airflow transition in an upward pattern from the tank, into the top of the unit, and with subsequent re-distribution into the room.

Investigators performed rounds on relevant inpatient units, perioperative areas, and operating rooms (ORs) to evaluate clinical workflows and adherence to tap water avoidance protocols as defined by Baker et al.^7^ Environmental samples of biofilms from various water sources and equipment requiring water for operation were obtained. In total, 5 distinct sites on each HCD were sampled, including the foam gasket, intake fan, main hose connector, cardioplegia hose connector, and bottom drain (Fig. 1). Biofilms from the distal water outlet, floor drain, and drainage equipment in the HCD cleaning and disinfection room were also sampled.

Qualitative airflow testing was performed using smoke to evaluate the potential for aerosolization of contaminated water through the device’s exhaust vent or any other unsealed openings.^5^ Two different simulations using micropowder smoke tests were performed while the device was running in normal operating mode^8^: (1) hinged lid closed and (2) hinged lid open (Fig. 1).

During the investigation, CardioQuip recommended the addition of airflow redirection shields on condenser unit exhaust fans on July 30, 2021.^9^ This modification was intended to redirect exhaust towards the floor and away from the patient and was implemented immediately.

### Laboratory methods

Standard mycobacterial culture methods were utilized for all clinical samples. Environmental samples were obtained using flocked swabs and streaked onto Middlebrook 7H11/7H11 selective media. Colonies suspicious for NTM underwent acid-fast staining and matrix-assisted laser desorption ionization-time of flight mass spectrometry (MALDI-TOF MS). A subset of clinical cultures with positive growth for *M. abscessus* was sent to Mycobacteria/Nocardia Research Laboratory at the University of Texas Health Science Center in Tyler, Texas, to identify the subspecies. Multilocus-sequence typing was performed using erythromycin ribosomal methylase (*erm(41)*) and region V RNA polymerase subunit β (*rpoβ*) gene sequencing.^10,11^ Variable-number tandem repeats (VNTRs) and pulsed-field gel electrophoresis (PFGE) were also performed on selected patient and environmental *M. abscessus* isolates.^12^ We compared *M. abscessus* isolates from the 2013–2015 outbreak, samples from 2018 (control), and samples from the current cluster using the same methods as Baker et al.^2^

## Results

### Epidemiologic investigation

**Figure 2.**
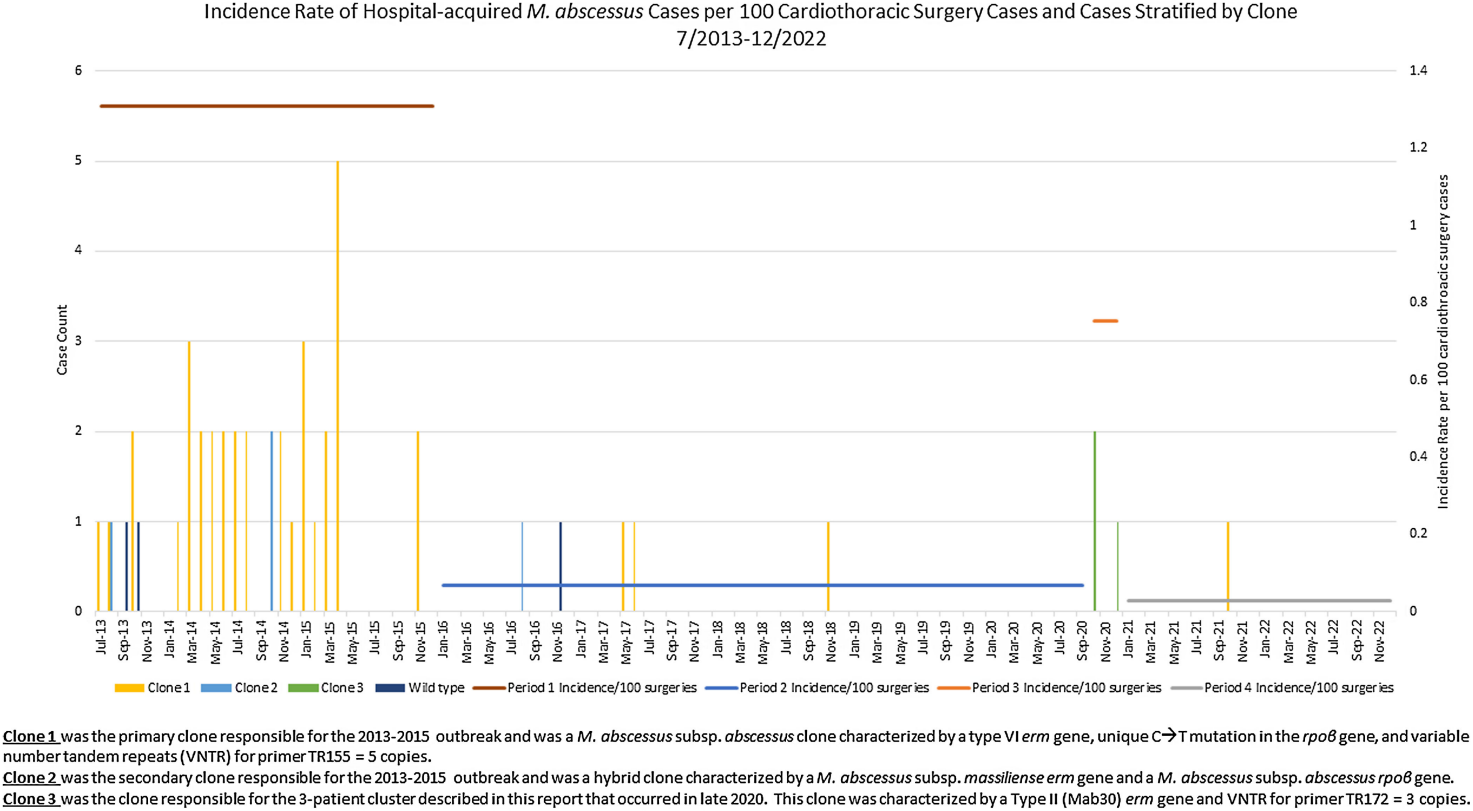
Incidence case count and incidence rates of hospital-acquired Mycobacterium abscessus extrapulmonary infections that occurred after cardiothoracic surgeries performed during the outbreak period, period 1 (7/2013-12/2015), post-outbreak period, period 2 (1/2016-9/2020), cluster, period 3 (10/2020-12/2020), and post-cluster, period 4 (1/2021-12/2022). Cases are stratified by clone of M. abscessus determined by molecular fingerprinting.

Between July 2013 and December 2022, 14,037 CT surgeries that commonly require a HCD (eg, heart and lung transplant, ventricular assistive device (VAD) placement, and cardiac bypass surgery) were performed at DUH, 48 of which were complicated by extrapulmonary Mab infection (Fig. 2). Most infections (81%) occurred during the previously described outbreak (period 1): incidence rate 1.31 cases per 100 CT surgeries (Fig. 2). In periods 2, 3, and 4 combined, 9 patients developed extrapulmonary Mab infections: incidence rate, 0.08 cases per 100 CT surgeries. Additional details regarding each of these 9 patients are outlined in Table 1. The incidence rate of HA-Mab infections was significantly lower during the postoutbreak period (period 2) and the postcluster period (period 4) compared to the cluster period (period 3) (Table 2).

**Table 1.** Description of 9 Cardiothoracic Surgery Patients With Suspected Hospital-Acquired *M. abscessus* in Periods 2, 3, and 4 (January 2016–December 2022)

Case	Type of Surgery	Culture Source	Period	Molecular Epidemiology Results^a^	Suspected Route/Mode of Transmission^b^
1	Lung transplant	Tissue	2	Clone 2 (subspecies massiliense)	Possible tap water exposure during patient bathing
2	ECMO	Blood	2	Wild type	Present on Admission
3	Right VAD removal	Blood	2	Clone 1 (subspecies abscessus, erm VI/ VNTR TR172 copy 2)	Possible tap water exposure during patient bathing
4	Lung transplant	Blood and BAL	2	Clone 1 (subspecies abscessus, erm VI/ VNTR TR172 copy 2)	Possible tap water aspiration leading to pulmonary infection (positive bronchoalveolar lavage)
5	Lung transplant	Tissue	2	Clone 1 (subspecies abscessus, erm VI/ VNTR TR172 copy 2)	Possible tap water exposure of ECMO cannulation site during patient bathing
6	Left VAD insertion	Tissue	3	Clone 3 (subspecies abscessus, erm Type II(Mab30)/ VNTR TR172 copy 3)	Probable intraoperative HCD-related transmission
7	Heart transplant	Tissue	3	Clone 3 (subspecies abscessus, erm Type II(Mab30)/ VNTR TR172 copy 3)	Probable intraoperative HCD-related transmission
8	Lung transplant	Blood	3	Clone 3 (subspecies abscessus, erm Type II(Mab30) VNTR TR172 copy 3)	Probable intraoperative HCD-related transmission

Note. ECMO, extracorporeal membrane oxygenation cannulation; VAD, ventricular assist device; HCD, heater-cooler device.

a Clone 1 was the primary clone responsible for the 2013–2015 outbreak and was a *M. abscessus* subsp *abscessus* clone characterized by a type VI *erm* gene, unique C◊T mutation in the *rpoβ* gene, and variable number tandem repeats (VNTR) for primer TR155 = 5 copies. Clone 2 was the secondary clone responsible for the 2013–2015 outbreak and was a hybrid clone characterized by a *M. abscessus* subsp *massiliense erm* gene and a *M. abscessus* subsp *abscessus rpoβ* gene. Clone 3 was the clone responsible for the 3-patient cluster described in this report that occurred in late 2020. This clone was characterized by a Type II (Mab30) *erm* gene and VNTR for primer TR172 = 3 copies.

b Possible vs probable designation relates to combination of clinical and epidemiologic data.

**Table 2.** Description of Outbreak and Postoutbreak Periods With Incidence Rates and Incidence Rate Ratio Comparisons of Hospital-Acquired *M. abscessus* Cases in Cardiothoracic Surgeries Between July 2013 and December 2022

Period	Date Range	No. of Hospital-Acquired Extrapulmonary *M. abscessus* Cases Detected after Cardiothoracic Surgery	No. of Cardiothoracic Surgeries^a^	Rate of Hospital-Acquired *M. abscessus* Cases per 100 Cardiothoracic Surgeries	Incidence Rate Ratio	*P* Value
1 (Outbreak)	7/2013–12/2015	39	2982	1.31	1.74	.35
2 (Post-outbreak)	1/2016–9/2020	5	7209	0.07	0.09	<.01
3 (Cluster)	10/2020–12/2020	3	398	0.75	1.00 (ref)	…
4 (Post-cluster)	1/2021–12/2022	1	3448	0.03	0.04	<.01

a Cardiothoracic surgeries included lung transplantation, heart transplantation, ventricular assistive device implantation, and cardiac bypass surgery.

Of the 3 CT surgery patients with postoperative *M. abscessus* infections during the cluster (period 3), 2 patients had transplant surgeries (1 lung, 1 heart), and 1 patient had a left VAD implanted. Of these 3 patients, 2 had surgery on the same day by the same surgeon. However, the surgeries were performed in different operating rooms, used distinct HCDs and bypass machines, and had separate perfusionist teams. The third case had surgery performed ∼6 weeks later, with a different surgeon, HCD, bypass machine, and perfusionist, but was performed in the same operating room as 1 of the first 2 cases. All case patients were cared for in the CT intensive care unit (ICU) following the operations. Two cases had *M. abscessus* isolated from tissue obtained from CT surgical sites and the third from the bloodstream. Patients were seen by infectious diseases specialists and were treated with combination antimicrobial therapy. Of the 3 patients, 2 subsequently died, largely related to their severe underlying comorbid conditions.

### Field investigation

Environmental samples that yielded positive cultures for *M. abscessus* included biofilm from 8 of the 12 HCDs, the plumbing proximal to the 0.2-µm Pall filter-outfitted water outlet used to fill HCDs, and the floor drain in the HCD cleaning room.

Of the initial 3 HCDs that we sampled, *M. abscessus* grew from 2 of the 3 devices, and *M. immunogenum* grew from the third device. We immediately removed these 3 HCDs from service and sequestered the devices. Among the remaining fleet of 9 HCDs, we cultured 3 HCDs per week, sampling 5 sites per device. In total, 10 (83%) of 12 HCDs were colonized with *M. abscessus* and 2 (17%) of 12 were colonized with *M. immunogenum*. *M. abscessus* was most commonly recovered from the cardioplegia connector site (4 of 12) followed by the foam gasket (3 of 12), main hose connector (2 of 12), and bottom drain (2 of 12). The fleet of HCDs was linked to *M. abscessus* transmission events within just 28 months of acquisition and deployment.

Review of the cleaning and maintenance logs for the HCD fleet as well as filter changes for the 0.2-µm filter on the outlet used for cleaning and filling the devices demonstrated strict adherence to all protocols. Observations of multiple CT surgery cases did not identify any significant opportunities for other sources of intraoperative contamination with water or other bioaerosols.

Care providers identified the following opportunities for improved adherence to the tap water avoidance protocol: patients may receive tap water from visitors in the form of ice chips, mouth moistening swabs, or water and patients may be exposed to tap water during bathing.

Smoke testing demonstrated concern for aerosolization as follows. In the first smoke testing scenario, micro puff smoke was applied at various locations around the top of the unit, resulting in an airflow pattern where air would enter the unit from the “front” (near the water add/check door) and would then be pushed out of the unit into the room. In the second scenario, with the water add/check door open, the micro puff smoke was applied at various locations around the top of the unit as well as from inside the water tank. The airflow patterns during this test were similar to those in the first scenario; however, when smoke was generated in the tank, it transitioned in an upward pattern from the tank, flowed into the condenser unit intake fan, and was redistributed throughout the room from the condenser exhaust fans (Fig. 1).

### Molecular epidemiology results

The 3 clinical isolates from period 3 were confirmed to be *M. abscessus* subspecies *abscessus* with Mab30 (type II) *erm* ^12^ and VNTR for primer TR172 = 3 copies (clone 3) (Table 1). Additionally, 2 out of 4 HCD isolates were confirmed to have the same *erm* gene and VNTR for TR172. The other 2 environmental isolates of *M. abscessus* (floor drain and upstream from water filter) represented a different clone with the same *erm* gene (type VI). VNTR for primer TR155 (5 copies) was the primary clone responsible for the 2013–2015 outbreak. A culture from 1 HCD grew *M. immunogenum*.

The PFGE patterns for the clinical and environmental isolates from this cluster differed by 0 or 1 band and were considered indistinguishable/clonal.^13^

### Mitigation strategy

A multifaceted mitigation strategy was implemented to reduce the risk of both HCD-related and non–HCD-related Mab exposure in the healthcare setting as follows. First, the entire fleet of HCDs was replaced given the high rate of colonization detected during environmental sampling. The same model of HCD was chosen due to limited options and availability of HCDs for purchase at the time of the cluster. Second, the engineering teams embarked on a longer-term path toward HCD containment and sequestration versus bioaerosol containment and HEPA filtration because the CT OR configurations did not allow HCDs to be placed outside the operating room and still maintain heating/cooling functionality. Complete containment of the device within the operating room space proved not to be a viable strategy due to impact on device functionality. Efforts for bioaerosol containment and HEPA filtration are currently ongoing. In addition to exploring additional engineering controls, the perfusion and clinical engineering teams continued all cleaning, disinfection, and maintenance protocols. Finally, the team remains vigilant for updates and/or new guidance put forth by the device manufacturer or FDA, allowing for immediate implementation of any new recommendations that could further mitigate risk.

Although this clonal cluster was not felt to be related to direct tap water exposure during hospitalization, investigators used the opportunity to improve adherence to tap water avoidance protocols as sporadic cases of HA-Mab infections from distinct *M. abscessus* clones were detected following the prior outbreak. A tap-water avoidance reeducation campaign was undertaken that included creation and multimodal dissemination of educational documents and presentations for staff, patients, family members, and visitors, including discharge instructions on tap water avoidance. Furthermore, an automated order based on patient location and/or surgery type was developed and embedded into the electronic medical record.

Investigators notified both the HCD manufacturer and the FDA of this cluster of infections and, to date, have not identified additional cases of extrapulmonary HA-Mab infections related to clone 3. The FDA responded to this notification with a public communication on September 30, 2022^14^ and had no additional recommendations for the investigation or mitigation response.

### Discussion

We investigated and mitigated a cluster of 3 extrapulmonary HA-Mab infections among CT surgical patients linked to a water-based HCD that was not previously associated with NTM outbreaks in the published literature. Despite rigorously adhering to the manufacturers IFU for strict use of filtered water, performing cleaning and disinfection more frequently than required, and following the recommended maintenance schedule, the HCD fleet became contaminated with *M.* abscessus within 28 months of acquisition. Rigorous surveillance and clinical and molecular epidemiology findings alerted the infection prevention team to this clonal cluster. The primary mitigation strategy included replacing the entire HCD fleet, allowing a multidisciplinary team to explore options for containment and/or HEPA filtration of bioaerosols.

Clone 3 of *M. abscessus* subsp *abscessus* responsible for this cluster of 3 extrapulmonary infections most likely originated from the municipal water supply. Based on the absence of other FDA Med Watch Mab reports associated with this device, the possibility of HCD contamination prior to arrival at our facility is low. It is likely that clone 1, responsible for the prior (2013–2015) HCD-associated outbreak at our hospital outcompeted clone 3 in the sampled plumbing biofilms, decreasing the likelihood of recovering clone 3. Although clone 3 was not isolated from any environmental samples aside from the HCDs, this clone has subsequently been isolated from 1 bronchoalveolar lavage (BAL) sample following replacement of the HCD fleet. Isolating clone 3 from BAL samples supports the presence of this clone in hospital tap water, and therefore, municipal water supply.

Although air sampling was not performed, internal smoke testing and published smoke testing data from LivaNova 3T HCDs supports aerosolization as the most likely primary mode of transmission.^15^ The decision to not perform air sampling during the investigation was made based on published studies supporting this mode of transmission, epidemiologic evidence from the internal investigation, and lack of appropriate controlled experimental space. Indirect transmission via the healthcare worker hands was felt to be less likely based on the lack of surgical team interaction with HCDs or perfusionist interaction with patients during CT surgeries.

Current interventions designed to prevent new cases of NTM infections linked to water-based HCDs are unrealistic, are under investigation, or lack the test of time. Use of sterile water, highly filtered water, and/or containment devices as a means to prevent HCD-related NTM infections are the primary strategies that have been proposed.^16^ Unfortunately, strict use of sterile water in HCDs is unrealistic for high-volume surgical centers given the frequency of supply chain shortages, and breakthrough NTM infections such as the cluster described in this report have occurred despite use of filtered water.^17^ Currently no exhaust containment device for HCDs exists. In addition, the impact of airflow redirection shields developed for the CardioQuip-m1000(i) on HA-NTM infections has not yet been quantified. Some institutions have isolated HCDs from the surgical field or operating room completely to reduce the risk of transmission from HCD exhaust bioaerosols, resulting in a successful termination of HCD-associated NTM infections.^17,18^ However, removal of the HCD from the surgical field requires reconfiguration of the operating room, which may not be possible due to impaired functionality of the HCD when placed too far from the patient.^19^ The cost and waste associated with replacing HCDs every few years to mitigate the potential for NTM colonization and transmission is not environmentally or economically sustainable.

Recently, a water-free HCD that uses a novel glycol-based thermal transfer technology entered the market. As part of the FDA clearance process, the device went through rigorous laboratory testing to evaluate for evidence of NTM aerosolization and ultimately was cleared for use in May 2022. However, real-world experience using the device under nonexperimental conditions is currently lacking.

In addition to evidence-based and time-tested risk mitigation strategies for HCD use, further guidance is needed on the optimal way to perform NTM surveillance and systematically report cases to better understand the true burden of HCD-related (and other) HA-NTM infections. NTM surveillance is highly resource-intensive and often requires the use of electronic microbiology reports, expert review of potential cases, access to molecular epidemiology analyses, and time-consuming field investigations. For example, a recent publication identified a cluster of *M. abscessus* infections in cardiac surgery patients and outlined the extensive investigation required to identify and mitigate the source.^20^ Heavily colonized ice machines on the cardiac unit were deemed to be the source of infection among CT surgery patients with bloodstream, sternal wound, and VAD driveline infections. Many hospitals likely lack sufficient support and infrastructure to create and sustain a rigorous surveillance and response system. Furthermore, standardized case definitions are not commonly used, and most states do not require NTM reporting. Therefore, the true incidence of HA-NTM infectious is likely higher than currently reported in the literature.

Our study had several limitations. First, we did not perform air sampling and thus we were unable to confirm that the aerosolization of mycobacteria was the definitive route of transmission for the 3 patients with extrapulmonary HA-Mab infections. However, given the results of the smoke testing, epidemiologic data from this investigation, and prior NTM outbreaks related to HCDs, aerosolization from HCDs was felt to be the most likely mode of transmission.^21^ In addition, HA-Mab surveillance was limited to patients presenting to our organization. Finally, it was not possible to definitively determine the mechanism of HCD inoculation with the *M. abscessus* clone responsible for this cluster. Though HCD contamination could have taken place prior to arrival at our facility, this seems unlikely for the aforementioned reasons.

We report a clonal cluster of 3 extrapulmonary HA-Mab infections among CT surgery patients linked to water-based HCDs. Although device replacement successfully mitigated the cluster, we are actively reviewing and planning longer-term mitigation strategies. We encourage other organizations to maintain a robust NTM surveillance system and to report cases associated with medical devices so that the FDA and manufacturers can continue to advance safety through design modifications and new technology.
